# Enterovirus 71 Induces INF2 Cleavage via Activated Caspase-2 in Infected RD Cells

**DOI:** 10.3389/fmicb.2021.684953

**Published:** 2021-05-11

**Authors:** Bei Wang, Chongyang Zhang, Congci Yu, Yue Zhu, Qing Tang, He Huang, Zhendong Zhao

**Affiliations:** ^1^National Health Commission (NHC) Key Laboratory of Systems Biology of Pathogens, Institute of Pathogen Biology, Chinese Academy of Medical Sciences and Peking Union Medical College, Beijing, China; ^2^Clinical Immunology Center, Chinese Academy of Medical Sciences & Peking Union Medical College, Beijing, China

**Keywords:** EV71, INF2, caspase-2, mitochondrial, viral replication

## Abstract

Enterovirus 71 (EV71) is the major causative pathogen of hand, foot, and mouth disease. The lack of understanding of the virus’s pathogenesis hinders the development of anti-virus drugs and the control of EV71 infection. Our previous studies have demonstrated that both mitochondria and endoplasmic reticulum (ER) were altered significantly in EV71 infected cells, but the mechanism is still unclear. In this study, we investigated the effects of EV71 infection on the expression of INF2, a key regulator factor in ER-Mitochondria communication and mitochondrial fission. We found that INF2 was cleaved in EV71 infected RD cells. The INF2 cleavage occurred at Aspartic 1,051 of INF2 and is mediated by activated caspases, predominantly by activated caspase-2. The subcellular localization of INF2 and caspase-2 was significantly altered in infected cells. We speculate that caspase-2-mediated INF2 cleavage is involved in forming viral replication organelles (ROs) and is a positive feedback regulatory mechanism of mitochondrial disorders caused by EV71 infection.

## Introduction

Enterovirus71 (EV71), which belongs to the *Enterovirus* genus of the Picornaviridae family, is the causative agent of hand, foot, and mouth disease (HFMD) and is especially the major cause of severe HFMD (Solomon et al., 2010). Acute EV71 infection can also lead to severe neurological manifestations and occasionally cause permanent paralysis or death. Since the first report in the United States in 1974 (Schmidt et al., 1974), EV71 outbreaks have been reported worldwide. In China, in 2008, the EV71 infection caused a serious HFMD epidemic in Fuyang City, Anhui Province. Since then, the frequency and severity of HFMD have shown an increased annual trend and pose a serious threat to children’s health (Zhang et al., 2010). The mechanisms underlying EV71 pathogenesis are unclear, and a lack of understanding of its viral pathogenesis does not allow the development of drugs against this virus and the control of EV71 infection (Solomon et al., 2010). Therefore, further research is needed to elucidate the pathogenesis of EV71.

Inverted formin 2 (INF2) is a formin protein that can polymerize and depolymerize actin filaments. Mutations in INF2 are associated with the neuropathy Charcot-Marie-Tooth disease and kidney disease focal and segmental glomerulosclerosis (Labat-de-Hoz and Alonso, 2020). There are two isoforms of INF2 in mammalian cells: the prenylated (CAAX) isoform and the non-CAAX isoform. These two isoforms differ in the C-terminal amino acid sequence and are different in cellular localization and function (Ramabhadran et al., 2011). The CAAX isoform of INF2 is peripherally bound to the cytoplasmic leaflet of the ER (Chhabra et al., 2009). It plays an important role in mitochondrial fission (Korobova et al., 2013; Manor et al., 2015), ER-to-mitochondrial calcium transfer, inner membrane constriction, and mitochondrial division (Jin et al., 2017; Chakrabarti et al., 2018); The INF2-non-CAAX isoform is loosely associated with a web-like meshwork in the cytoplasm and plays an important role in the maintenance of Golgi architecture (Ramabhadran et al., 2011).

Our previous studies have demonstrated that both mitochondria and endoplasmic reticulum (ER) were altered significantly in EV71 infected cells, and EV71 may use ER-derived membranes to form viral replication organelles (ROs) to support replication (Wang et al., 2013, 2017). However, what mechanisms drive this change is still unclear. This study found that INF2, a key factor in ER-Mitochondria communication and mitochondrial fission, is cleaved in EV71 infected RD cells. The cleavage of INF2 occurs at Aspartic 1,051 and is mediated by activated caspases, predominantly by activated caspase-2. The caspase-2-mediated INF2 cleavage may be involved in the formation of viral ROs and virus-induced mitochondrial disorders.

## Materials and Methods

### Cells and Viruses

Rhabdomyosarcoma (RD) cells were purchased from the ATCC. It was cultured in MEM (modified Eagle’s medium) supplemented with 10% fetal bovine serum (FBS) and 100 U/mL penicillin and 0.1 mg/ml streptomycin. BSRT7 cells were described in our previous study (Wang et al., 2017) and were cultured in DMEM (Dulbecco’s modified Eagle’s medium) supplemented with 10% FBS and 1 mg/mL G418. EV71 is a Fuyang strain (GenBank accession no. FJ43976 9.1), propagated in RD cells.

### Antibodies and Reagents

The antibodies used in this study were as follows: INF2 (1:500, Proteintech, catalog number 20466-1-AP), caspase-2 (1:500, Proteintech, catalog number 10436-1-AP), caspase-3 (1:500, Proteintech, catalog number 19677-1-AP), caspase-4 (1:500, Proteintech, catalog number 11856-1-AP), caspase-6 (1:500, Proteintech, catalog number 10198-1-AP), caspase-8 (1:500, Proteintech, catalog number 13423-1-AP), caspase-10 (1:500, Proteintech, catalog number 14311-1-AP), caspase-7 (1:1,000, Cell Signaling Technology, #9492), PARP (1:1,000, Cell Signaling Technology, #9542), β-actin (1:5,000, Sigma, A1978), GFP (1:2,000, Sigma, G1546), Flag (1:2,000, Sigma, F1804), V5 (1:1,000, Sigma, V8012), and EV71 VP1 (1:1,000, Abnova, MAB1255-M05). The corresponding IRDye 680- or 800- labeled secondary antibodies were obtained from LI-COR Biosciences. The fluorescence-labeled secondary antibodies used in immunostaining were purchased from Jackson ImmunoResearch. EV71 2C antibodies were generated in mice using recombinant protein as the immunogen. Z-VAD-FMK, Z-DEVD-FMK, and Z-IETD-FMK were all purchased from Selleck. Z-VDVAD-FMK was purchased from Biovision.

### Transfection of Plasmids and siRNAs

Cells were transfected with plasmids or siRNA duplexes using Lipofectamine 3000 (Invitrogen, L3000015) and Lipofectamine RNAiMAX (Invitrogen, 13778100) according to the manufacturer’s instructions, respectively. siRNAs were purchased from Guangzhou RiboBio and transfected at a final concentration of 40 nM.

### Cell-Free Caspases Cleavage Assay

Human activated caspases were purchased from Enzo Life Sciences (catalog number BML-AK010) and used at a final concentration of 5 U/μL. In each assay, cellular protein extracts (100 μg) were incubated with active caspase at 37°C for 2 h in reaction buffer (50 mM HEPES (pH 7.5), 100 mM NaCl, 1 mM EDTA, 0.5% CHAPS, 10 mM DTT).

### Colorimetric Caspase Activity Assay

According to the manufacturer, caspase activities from mock- and EV71-infected RD cells were measured with the Caspase Colorimetric Substrate Set II Plus kit’s instructions (Biovision, catalog number K138-9-25). Briefly, cellular protein extracts (100 μg) collected from cells were added to a reaction buffer containing a *p*-nitroanilide-labeled specific caspase substrate and incubated for 2 h at 37°C. OD405 was used to measure the relative caspase activity. Results are presented as mean ± SD of three replicates.

### Generation of Gene Knockout Cell Lines by CRISPR-Cas9 System

The lentiCRISPR v2 plasmid containing the corresponding sgRNA was transfected into RD cells to generate gene knockout cell lines. Cell clones with the desired gene knockout were first screened by immunoblot analysis and then confirmed by sequencing the gene’s PCR products. The sgRNA sequences used in this study are as follows.

sgRNA for INF2: TCCGTGGGGTCCGAATCCTG
sgRNA for caspase-2: AAAGAACTGGAATTTCGCTC
sgRNA for caspase-3: GGAAGCGAATCAATGGACTC

### Immunofluorescence and Confocal Microscopy

The RD cells were fixed with 4% paraformaldehyde in PBS for 15 min and permeabilized using 0.25% TritonX-100 in PBS for 15 min. After washing three times with PBS, the cells were blocked with 5% BSA (Sigma, A7906) for 1 h and then incubated with primary antibodies overnight at 4°C. The following day, cells were washed three times and incubated with the appropriate fluorescence-conjugated secondary antibodies (Jackson Immuno Research) for 1 h at room temperature, followed by nuclear staining with DAPI. Images were captured using a TCS SP5 laser-scanning confocal microscope (Leica Microsystems).

## Results

### EV71 Induces INF2 Cleavage in Infected Cells

**FIGURE 1:**
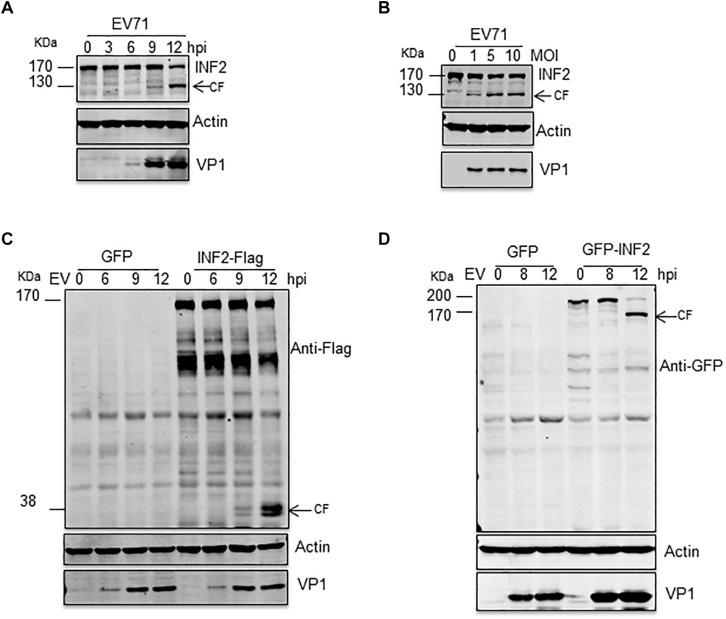
EV71 induces INF2 cleavage in infected cells. **(A)** RD cells were infected with EV71 (MOI = 10) for the indicated times (hpi, h post-infection), and **(B)** RD cells were infected with the suggested dosage of EV71. The cells were then harvested, and Western blotting was performed using the indicated antibodies to detect INF2, EV71 VP1, and Actin. Arrows indicate the cleavage fragments (CF). **(C,D)** RD cells were transfected with plasmids encoding GFP control/INF2-Flag **(C)** or GFP control/GFP-INF2 **(D)**, 24 h after transfection, the cells were infected with EV71 (MOI = 10) for the indicated times (hpi, h post-infection), and then the cells were harvested, and Western blot was performed using the indicated antibodies. Arrows indicate the cleavage fragments (CF).

INF2 is a formin protein that can regulate both the polymerization and depolymerization of actin filaments. Recently, INF2 was identified as a key regulatory factor of mitochondrial fission, ER-Mitochondria calcium transfer, mitochondrial inner membrane constriction, and division (Korobova et al., 2013; Chakrabarti et al., 2018). Since both the mitochondria and ER were altered significantly in EV71 infected cells, we hypothesized that viral infection would affect INF2 expression. Time- and dose-dependent studies were conducted, and the expression of INF2 in EV71-infected RD cells was determined by Western blot assay. The results showed that the cleavage of INF2 induced by EV71 was time-and dose-dependent. The full-length INF2 was detected with an apparent molecular mass of ∼170 kDa. The cleavage band was detected at ∼130 kDa. The cleavage band’s intensity increased when the infection proceeded or the viral dosage increased (Figures 1A,B). To further locate the cleavage site, RD cells were transfected with a plasmid encoding a C-terminal FLAG-tagged INF2 (INF2-Flag) or an N-terminal GFP-tagged INF2 (GFP-INF2). The cells were infected with EV71 for different times as indicated, and INF2 and its cleavage products were detected using antibodies against the Flag and GFP tags. As results showed, in RD cells transfected with INF2-Flag, cleavage fragments could be detected with a molecular weight of about 38 kDa using Flag antibody (Figure 1C), while in RD cells transfected with GFP-INF2, the cleavage fragments could be detected with a molecular weight of about 160 kDa by GFP antibody (Figure 1D). These results indicate that the cleavage site is near the C-terminal end of INF2, approximately at the amino acid domain 1,000–1,100 of INF2.

### Activated Caspases Mediate EV71-Induced INF2 Cleavage

**FIGURE 2:**
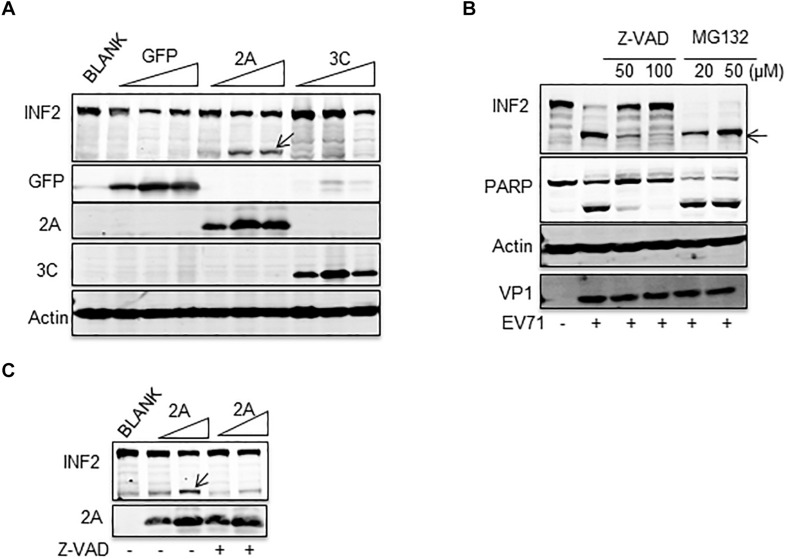
EV71-induced INF2 cleavage was mediated by caspase. **(A)** BSR/T7 cells were not transfected (BLANK) or transfected with increasing dosage of plasmids encoding GFP control, EV71 2A^pro^, and EV71 3C^pro^. Thirty-six h after transfection, the cells were lysed, and a Western blot was conducted to detect INF2, GFP, 2A^pro^, 3C^pro^, and actin. Arrows indicate the cleavage fragments (CF). **(B)** RD cells were mock-infected (–) or infected with EV71 (MOI = 10) for 12 h, Z-VAD-FMK (Z-VAD), and MG132 of different concentrations were added to the culture media 6 h post-infection. Then, the cells were harvested and lysed for Western blot detecting INF2, PARP, Actin, and EV71 VP1. PARP was used as an indicator of caspase activation and the effect of Z-VAD. Arrows indicate the cleavage fragments (CF). **(C)** BSR/T7 cells were not transfected (BLANK) or transfected with increasing dosages of plasmids encoding EV71 2A^pro^, 24 h after transfection; the cells were not treated (–) or treated with 100 μM Z-VAD for an additional 12 h, then the cells were lysed and Western blot was conducted to detect INF2 and 2A^pro^. Arrows indicate the cleavage fragments (CF).

To clarify the precise mechanism by which INF2 is cleaved, we first investigated whether EV71-encoded viral proteases 2A^pro^ and 3C^pro^ participate in this process. Plasmids encoding EV71 2A^pro^ and 3C^pro^ were transfected into BSR/T7 cells. These cells stably expressed T7 RNA polymerase and proved to be a successful model for expressing EV71 2A^pro^, inhibiting host genes’ expression (Wang et al., 2017). The results showed that cleavage fragment was only detected in cells expressing EV71 2A^pro^, but not in GFP control or EV71 3C^pro^-expressing cells, indicating that EV71 2A^pro^ participated the cleavage of INF2 (Figure 2A). In addition to viral-encoded proteases, activated caspases and proteasome are also characterized as the principal executors for cutting or degrading host factors in virally infected cells (Gao and Luo, 2006; Chen et al., 2017; Ning et al., 2019). To test the role of caspases and proteasome in cleavage of INF2, pan-caspases inhibitor Z-VAD-FMK and proteasome inhibitor MG132 were applied to treat EV71-infected RD cells. Z-VAD-FMK treatment completely inhibited INF2 cleavage at high concentration, while MG132 did not affect it (Figure 2B). These results indicate that the cleavage of INF2 results from activated caspases in infected cells. According to the above results, we speculate that activated caspases also mediate the cleavage of INF2 in EV71 2A^pro^ transfected cells. BSR/T7 cells transfected with the plasmid encoding EV71 2A^pro^ were also treated with Z-VAD-FMK, and INF2 cleavage was detected to test this hypothesis. As we expected, the Z-VAD-FMK treatment could also inhibit EV71 2A^pro^ induced INF2 cleavage (Figure 2C), indicating that EV71 2A^pro^ is responsible for caspase activation and activated caspases are the final players of INF2 cleavage in EV71 infected cells.

### INF2 Is Cleaved at Aspartic 1,051

**FIGURE 3:**
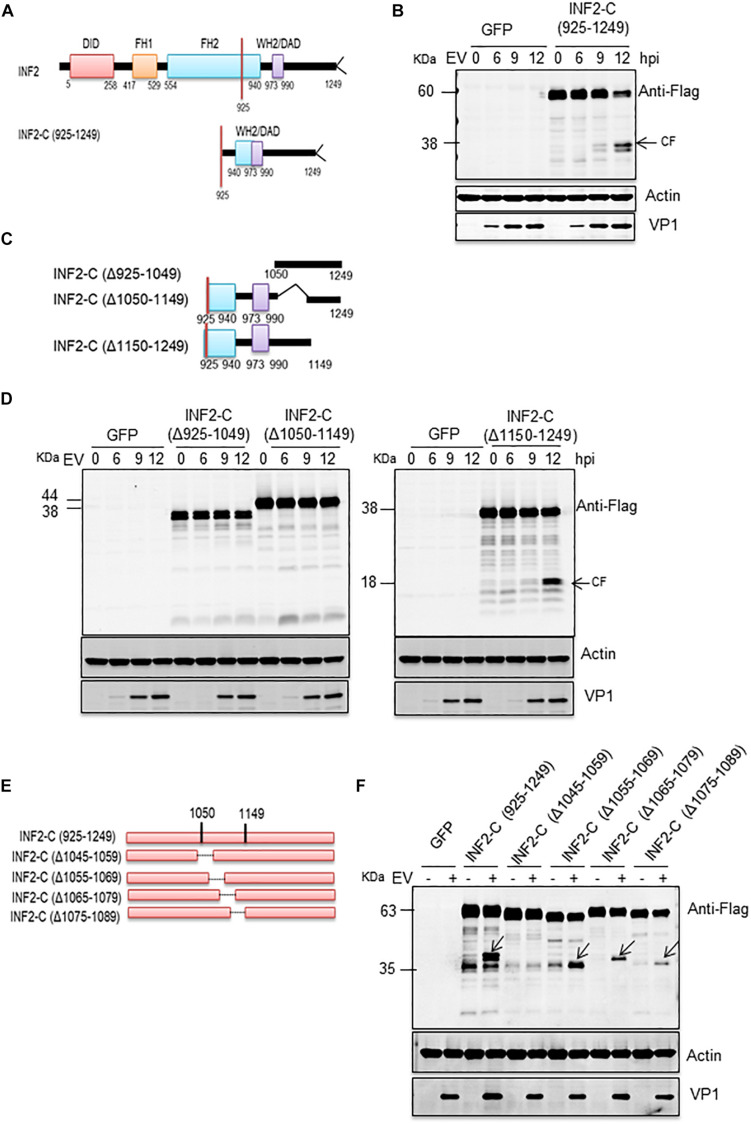
EV71-induced INF2 cleavage is located at the 1,045–1,059 amino acid region of INF2. **(A)** Schematic diagram of the INF2 structural domains. **(B)** RD cells were transfected with plasmids encoding GFP control or INF2-C (925–1,249), 24 h after transfection, the cells were infected with EV71 (MOI = 10) for the indicated times (hpi, h post-infection), and then the cells were harvested, and Western blot was performed using the indicated antibodies. Arrows indicate the cleavage fragments (CF). **(C)** Schematic diagram of deletion mutants sued in **(D)**. **(D)** RD cells were transfected with plasmids encoding the GFP control, INF2-C (Δ925–1,049), INF2-C (Δ1,050–1,149), and INF2-C (Δ1,150–1,249); then the cells were be treated as described in **(B)**. **(E)** Schematic diagram of deletion mutants sued in **(F)**. **(F)** RD cells were transfected with plasmids GFP control and indicated deletion mutants. The cells were then treated as described in **(B)**.

According to the above results, the cleavage site of activated caspases on INF2 may locate at the region of its amino acids (aa) 1,000–1,100. To precisely locate the cleavage site, we constructed a deletion mutant based on the INF2-Flag. The deletion mutant of the INF2 amino acids 925–1,249 [INF2-C (925-1249)] was predicted to still contains the potential cleavage site (Figure 3A). At first, a plasmid encoding INF2-C (925–1,249) was transfected into RD cells to assess whether EV71 infection could still induce its cleavage. As we expected, EV71 infection could induce INF2-C (925–1,249) cleavage, generating cleavage products with a molecular mass of ∼38 KDa, which could be detected by the Flag antibody and is the same molecular weight as the cleavage bands generated from INF2-Flag (Figure 3B), indicating that the cleavage site was located at the region of 925–1,249 aa and the cleavage product detected was the C-terminal cleavage fragments from INF2. Three rounds of screening were carried out using different deletion and point mutants based on INF2-C (925–1,249) to identify the cleavage site further.

In the first round of screening, three deletion mutants were constructed. Each contained a 100–125 aa deletion from INF2-C (925–1,249), namely INF2-C (Δ925–1049), INF2-C (Δ1,050–1,149), and INF2-C (Δ1,150–1,249) (Figure 3C). The results showed that cleavage products induced by EV71 could only be detected in INF2-C (Δ1,150–1,249) transfected cells (Figure 3D), which indicated that the cleavage site was located at the region of 925–1,149 aa. Since no cleavage products could be detected in both INF2-C (Δ925–1,049) and INF2-C (Δ1,050–1,149) transfected cells, we suspected the cleavage site might locate around the 1,050 aa so that the construction of deletion mutants destroyed the cleavage site or the cleavage products could not be discriminated from its parental deletion mutants since the molecular weight is very approximately with each other.

The second round of screening was conducted to confirm this speculation. The deletion mutants used in this round screening were INF2-C (Δ1,045–1,059), INF2-C (Δ1,055–1,069), INF2-C (Δ1,065–1,079), and INF2-C (Δ1,075–1,089); each one contained a 15-aa deletion ranging from 1,045 to 1,089 aa region of INF2 and was constructed based on INF2-C (925–1,249) (Figure 3E). As illustrated in Figure 3F, EV71-induced cleavage fragments could be detected in INF2-C (Δ1,055–1,069), INF2-C (Δ1,065–1,079), and INF2-C (Δ1,075–1,089) transfected cells but not in INF2-C (Δ1,045–1,059) transfected cells, demonstrating that the cleavage site is located in the region of INF2 1,045-1,059 aa.

**FIGURE 4:**
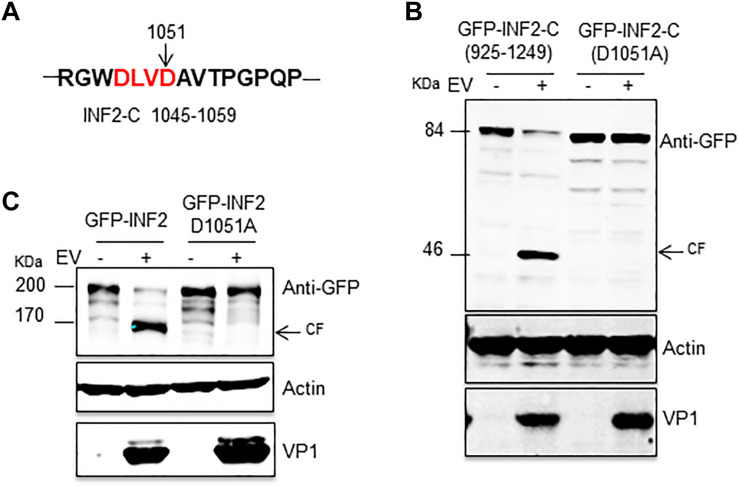
INF2 was cleaved at Aspartic 1,051 in EV71-infected cells. **(A)** Schematic diagram showing the sequences of 1,045–1,059 amino acids. The typical caspase cleavage residues are highlighted in red font. **(B)** RD cells were transfected with plasmids encoding GFP-INF2-C (925–1,249) or GFP-INF2-C (D1051A), 24 h after transfection, the cells were infected with EV71 (MOI = 10) for the indicated times (hpi, h post-infection), and then the cells were harvested, and Western blot was performed using the indicated antibodies. Arrows indicate the cleavage fragments (CF). **(C)** RD cells were transfected with plasmids encoding GFP-INF2 or GFP-INF2 (D1051A); then, they were treated as described in **(B)**.

In the third round of screening, we focused on the 1,045–1,059 aa of INF2 and found a typical caspase cleavage site, “DLVD,” which consists of the 1,048–1,051 aa (Figure 4A). Then we mutated Aspartic 1,051 to Alanine based on plasmid GFP-INF2-C (925–1,249) and transfected the plasmid [GFP-INF2-C (D1051A)] into cells to check whether the mutation destroyed the cleavage site on INF2. The results showed that the mutant was resistant to EV71-induced cleavage, demonstrating the activated caspases’ cleavage site on INF2 (Figure 4B). For further confirmation, the D1051A point mutation was also introduced into the GFP-INF2 plasmid encoding full-length INF2, and a similar result was obtained; The introduction of D1051A point mutation makes INF2 resistant to EV71 induced cleavage (Figure 4C). To verify that the cleavage is directly mediated by activated caspases and rule out the possibility that D1051A causes a conformational change to make INF2 resistant to caspase-2, the cleavage fragment in EV71-infected INF2-C (925–1,249)-transfected cells were isolated by immunoprecipitation (IP), and analyzed by N-terminal sequencing of first 10 amino acids. Although the sequencing results of the first five amino acids were ambiguous, which may be related to the impure products obtained by IP, the sequencing results of amino acids 6–10 were “PQPTL,” identical to INF2 amino acids 1,057–1,061, well demonstrated the cleavage occurred at Aspartic 1,051 (Supplementary Figures 1A,B). These results demonstrated that the Aspartic 1,051 on INF2 is the direct cleavage site of activated caspases.

### Multiple Caspases Were Activated in EV71-Infected Cells, of Which Caspase-2 Plays a Dominant Role in INF2 Cleavage

**FIGURE 5:**
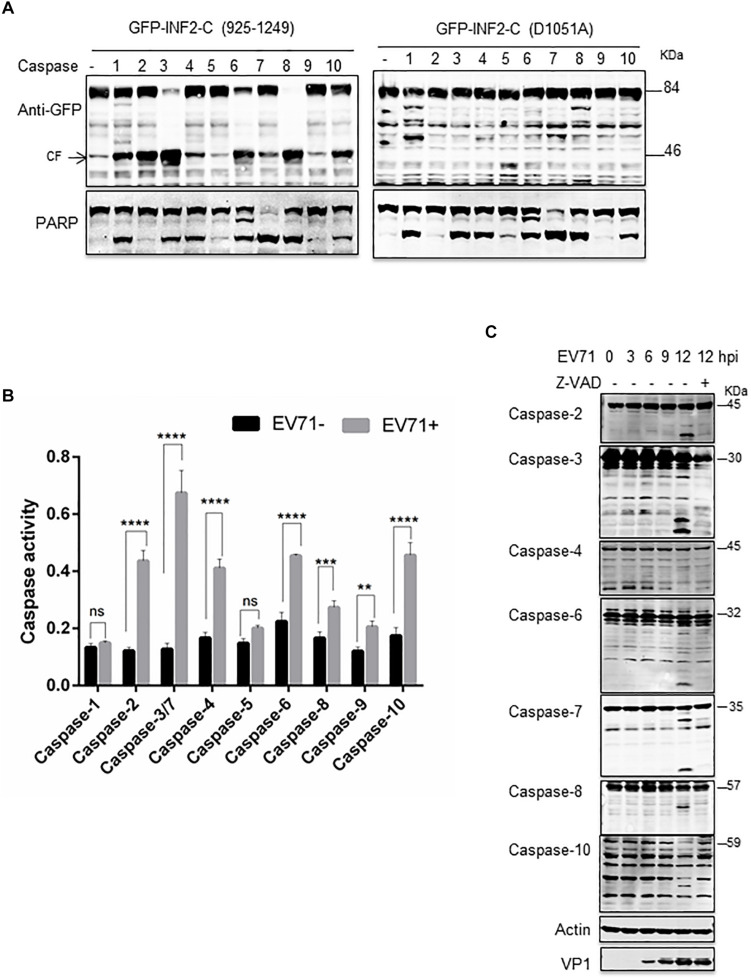
Multiple caspases are activated in EV71-infected cells. **(A)** *in vitro* caspases cleavage assay. RD cells were transfected with plasmids encoding GFP-INF2-C (925–1,249) or GFP-INF2-C (D1051A). Twenty-four h after transfection, the cells were lysed. The cell lysate was incubated with the indicated activated caspases for *in vitro* cleavage assay; then, a Western blot was conducted with the indicated antibody. PARP was used as an indicator of most caspases’ activation, and arrows indicate the cleavage fragments (CF). **(B)** RD cells were infected with EV71 (MOI = 10) for the indicated times, untreated (–) or treated (+) with 100 μM Z-VAD, for 6 h. The cells were harvested, and Western blot was performed using the indicated antibodies against different caspases, actin, and VP1. **(C)** Colorimetric caspase activity assay in mock-infected (EV71-) and EV71 infected RD cells. RD cells were infected with EV71 (MOI = 10) for 12 h, the cells were then harvested, and cell lysis was incubated with caspase colorimetric substrate to determine the caspase activity. Results are presented as mean ± SD of three replicates. Statistical significance was determined by the two-way ANOVA multiple comparisons test. ns, not significant. **P < 0.01; ***P < 0.001; ****P < 0.0001.

To further determine the caspases involved in EV71-induced INF2 cleavage, a cell-free cleavage assay was conducted with activated human caspases 1–10. Since the full-length INF2 is unstable under extracellular conditions, the cell lysate from cells overexpressing GFP-INF2-C (925–1,249) and GFP-INF2-C (D1051A) was used. The results showed that activated caspases-1, -2, -3, -4, -6, -7, -8, and -10 could cleave GFP-INF2-C (925–1,249) and produce a cleavage fragment with a molecular weight of ∼46 kDa, but caspases-5 and -9 could not. Moreover, different caspases cleave GFP-INF2-C (925–1,249) with different efficiencies, and the ∼46 kDa cleavage product could not be detected in the reaction with GFP-INF2-C (D1051A) overexpressed cell lysates (Figure 5A). Next, we assessed which caspases are activated among caspase-1 to caspase-10 in EV71-infected cells by colorimetric caspase activity assay. The result showed that caspases-2, -3, -4, -6, -7, -8, -9, and -10 were activated in EV71-infected cells compared with uninfected cells (Figure 5B). To confirm the activated status of the caspases mentioned above, Western blots were conducted to detect those caspases and their activated cleavage fragments. Meanwhile, EV71-infected cells treated with Z-VAD-FMK were taken as a negative control. As shown in Figure 5C, caspase-2, -3, -6, -7, -8, and -10 but not caspase-4 can be detected with obvious cleavage fragments, and these fragments disappeared when Z-VAD-FMK was added. These results confirmed the activated status of caspase-2, -3, -6, -7, -8, and -10 in EV71-infected cells (Figure 5C).

**FIGURE 6:**
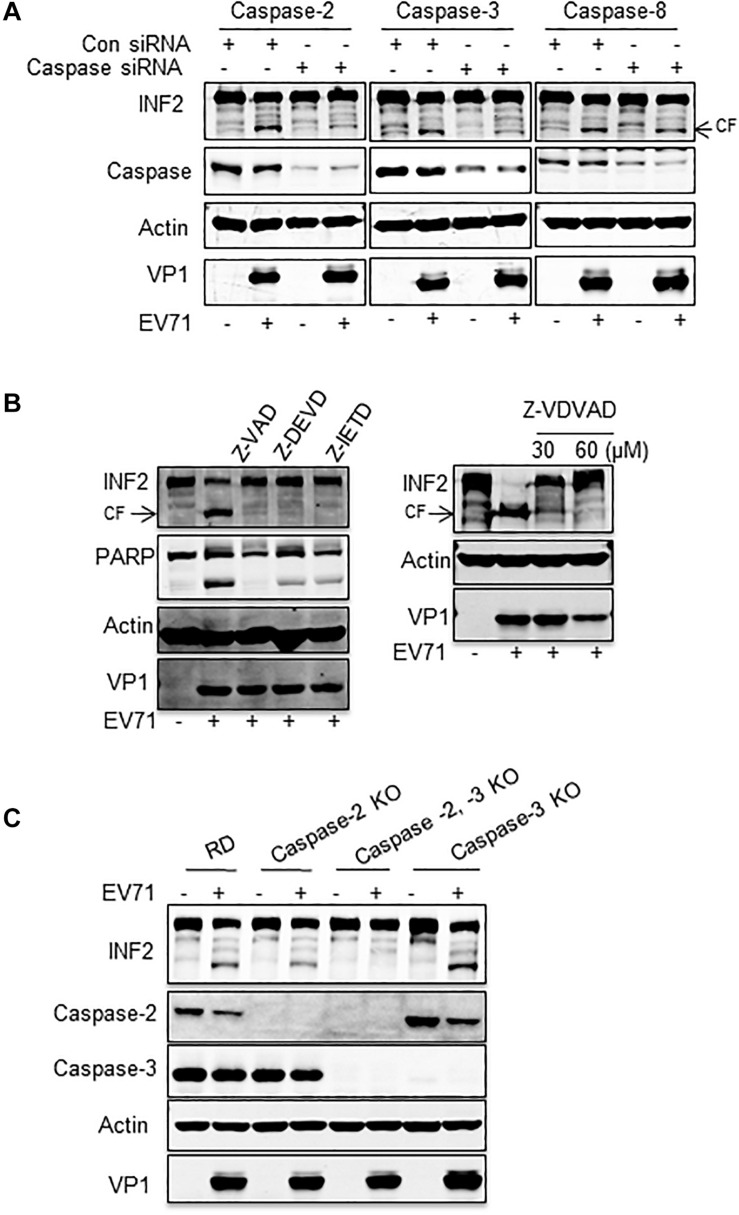
Caspase-2 plays a dominant role in EV71-induced INF2 cleavage. **(A)** RD cells were transfected with control siRNA or siRNA against caspase-2, -3, and -8, and 36 h after transfection, the cells were mock-infected (EV71-) or infected with EV71 (MOI = 10) for an additional 12 h. Then, the cells were harvested, and a Western blot was performed to detect INF2, the corresponding caspases, actin, and VP1. **(B)** RD cells were mock-infected (–) or infected with EV71 (MOI = 10) for 12 h, 100 μM Z-VAD-FMK (Z-VAD, pan-caspase inhibitor), Z-DEVD-FMK (Z-DEVD, caspase-3 inhibitor), Z-IETD-FMK (Z-IETD, caspase-8 inhibitor) (the left panel), and 30 or 60 μM Z-VDVAD-FMK (Z-VDVAD, caspase-2 inhibitor) were added to the culture media 6 h post-infection. Then, the cells were harvested and lysed for Western blot to detect INF2, PARP, Actin, and EV71 VP1. PARP was used as an indicator of caspase activation and the effect of caspase inhibitors. Arrows indicate the cleavage fragments (CF). **(C)** RD cells, caspase-2 knockout (KO) cells, caspase-2, -3 double KO cells, and caspase-3 KO were infected with EV71 (MOI = 10) for 12 h; then the cells were harvested, and Western blot was conducted to detect INF2, caspase-2, caspase-3, actin, and EV71 VP1.

Then, siRNA targeting caspase-2, -3, -4, -6, -7, -8, and -10 was used to silence the corresponding caspases, and EV71-induced INF2 cleavage was assessed in the siRNA-transfected cells. The results showed that the decrease of caspase-2, -3, and -8 inhibited INF2 cleavage in different degrees (Supplementary Figure 2 and Figure 6A), which indicated their role in EV71-induced INF2 cleavage. Next, caspases-2, -3, -8 inhibitors (Z-VDVAD-FMK for caspase-2; Z-DEVD-FMK for caspase-3; Z-IETD-FMK for caspase-8) were used to treat EV71-infected cells, and the pan-caspase inhibitor Z-VAD-FMK was used as the positive control, and the cleavage of INF2 was evaluated. However, the results showed that all these inhibitors could inhibit the cleavage of INF2 to a great extent, which indicates that these inhibitors have cross-reaction with each other and are not suitable for evaluating the effect of a single caspase (Figure 6B). Next, we knocked out certain caspase genes using CRISPR-Cas9 technology. Since caspase-2 and caspase-3 knocking down exhibit a relatively obvious effect on the inhibition of INF2 cleavage (Figure 6A), we first generated caspase 2 and/or 3 knockout cell lines. EV71-induced cleavage of INF2 was evaluated in these knockout cell lines. INF2 cleavage was completely inhibited in the caspase-2, -3 double-knockout cell line, and it was also greatly inhibited in the caspase-2 knockout cell line. Unexpectedly, in the caspase-3 knockout cell line, INF2 cleavage was not inhibited but was promoted (Figure 6C). Since the expression of caspase-2 is upregulated in the caspase-3 knockout cell line, we thought the increased INF2 cleavage was caused by the compensatory mechanism between caspase-2 and caspase-3.

The above results demonstrated that multiple caspases were activated in cells infected with EV71. Among these, both caspase-2 and caspase-3 participate in EV71-induced INF2 cleavage, and caspase-2 plays the dominant role.

### Caspase-2-Mediated INF2 Cleavage Participates in Viral Replication and Is the Cause of Mitochondrial Disorders

**FIGURE 7:**
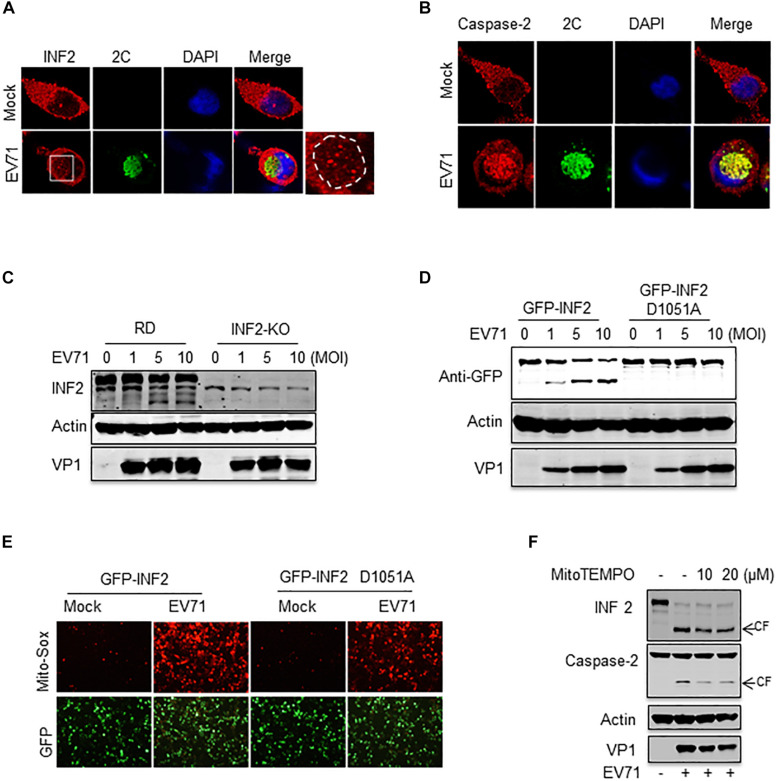
Caspase-2-mediated INF2 cleavage participates in viral replication and is the cause of mitochondrial damage. **(A)** RD cells were mock-infected or infected with EV71 (MOI = 10) for 12 h, and immunostaining was then performed to detect the intracellular distribution of INF2 and EV71 2C protein (INF2, red; 2C, green; nuclei, blue). Insets show magnified views of the red channel in the boxed region. **(B)** RD cells were treated as described in **(A)**, immunostaining was performed to detect the intracellular distribution of caspase-2 and EV71 2C protein (caspase-2, red; 2C, green; nuclei, blue). **(C)** RD cells and INF2 knockout RD cells (INF2-KO) cells were infected with the indicated dosage of EV71. The cells were then harvested, and Western blotting was performed using the indicated antibodies to detect INF2, actin and EV71 VP1. **(D)** INF2-KO RD cells were transfected with plasmids encoding GFP-INF2 or GFP-INF2 (D1051A). Twenty-four h after transfection, the cells were infected with the indicated dosage of EV71 and then harvested for Western blotting using the indicated antibodies. **(E)** INF2-KO RD cells were transfected with plasmids encoding GFP-INF2 or GFP-INF2 (D1051A). Twenty-four h after transfection, the cells were mock-infected or infected with EV71 (MOI = 10) for 12 h; then, the cells were stained with Mito-Sox (red) and photographed using a fluorescence microscope. **(F)** RD cells were mock-infected (EV71–) or infected with EV71 (MOI = 10) for 12 h in the absence of (–) or with MitoTEMPO (+) of the indicated concentrations. The cells were then harvested, and Western blotting was performed using the indicated antibodies to detect INF2, caspase-2, actin, and EV71 VP1. Arrows indicate the cleavage fragments (CF).

To evaluate whether EV71-induced INF2 cleavage is involved in viral replication, the subcellular localization of INF2 and caspase-2 in EV71-infected cells was first examined. EV71 2C protein was selected as a marker of viral replication organelles. Our previous study showed that EV71 2C was located in the perinuclear viral replication organelles and co-localized with host factors, including phosphatidylinositol4-kinaseIIIβ (PI4KB), ADP-ribosylation factor1 (ARF1), and p97 (Wang et al., 2013). INF2 exhibits almost evenly distributed signals in un-infected RD cells, but its distribution pattern changed significantly in EV71-infected cells. In the region where EV71 2C is located, the evenly distributed INF2 signals are attenuated and replaced by some large punctate structures (Figure 7A). As to the distribution of caspase-2, it also changed. It is distributed evenly in uninfected cells but redistributed to the perinuclear region, formed aggregation foci, and co-localized with EV71 2C in EV71 infected cells (Figure 7B). These results suggest that caspase-2-mediated INF2 cleavage may be involved in the formation of viral replication organelles.

To further assess the role of INF2 and INF2 cleavage in EV71 infection, an RD cell line with INF2 knockout was generated by CRISPR-Cas9 technology. Then, the cells were infected with EV71. The result showed that INF2 deficiency did not affect viral replication. EV71 viral protein was expressed equally in WT and INF2 knockout cells (Figure 7C). Next, plasmids encoding GFP-INF2 or cleavage-resistant point mutation GFP-INF2 D1051A were transfected into INF2 knockout cells to complementary expressed INF2 gene and then infected with EV71. The results showed that there were still no detectable differences in viral protein expression detected between the two groups (Figure 7D). These data suggest that caspase-2 mediated INF2 cleavage may play a redundant role in viral replication.

**FIGURE 8:**
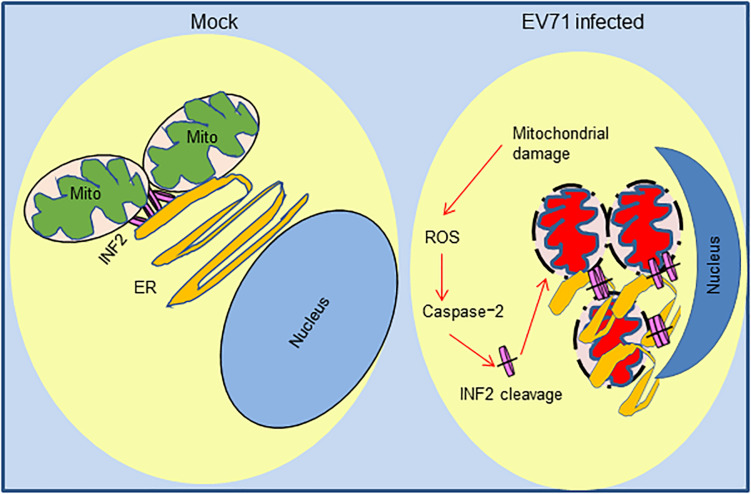
Positive feedback regulatory mechanism of caspase-2 mediated INF2 cleavage in EV71-induced mitochondrial damage. In normal cells, INF2 is located at the ER and regulates mitochondrial dynamics; In EV71-infected cells, mitochondrial and ER are damaged, and ROS of mitochondrial source activate caspase-2, which cleaves INF2 and exacerbates mitochondrial damage in turn.

Since INF2 was identified as a key regulatory factor of mitochondrial function and our previous study demonstrated EV71 caused mitochondrial disorders in infected cells, we assessed whether EV71-induced INF2 cleavage is related to mitochondrial disorders in infected cells. Plasmids encoding GFP-INF2 or GFP-INF2 D1051A were transfected into INF2 knockout cells, cells were then infected with EV71, and mitochondrial function was determined by mitochondrial reactive oxygen species (ROS) staining. ROS production was detected in EV71 infected cells, which indicated that EV71 caused mitochondrial damage. However, the ROS production in EV71-infected, GFP-INF2 D1051A transfected cells decreased (Figure 7E), demonstrating that caspase-2 mediated INF2 cleavage contributes to EV71-induced mitochondrial disorders. Since previous studies have demonstrated that caspase-2 could be activated by ROS and play an important role in stress-induced apoptosis, we speculate that caspase-2 mediated INF2 cleavage functions as a positive feedback regulatory mechanism in EV71-induced mitochondrial damage. To test our hypothesis, MitoTEMPO was used to eliminate ROS from the mitochondrial source. The results showed that both EV71-induced caspase-2 activation and INF2 cleavage were weakened in the cells treated with MitoTEMPO, as evidenced by the decreased intensity of cleavage fragments of INF2 and caspase-2 (Figure 7F), indicating that caspase-2 mediated INF2 cleavage is a positive feedback regulatory mechanism of EV71-induced mitochondrial damage (Figure 8).

## Discussion

Our study investigated the expression of mitochondrial regulator factor INF2 in EV71-infected cells. INF2 was cleaved at Aspartic 1,051 in infected cells, and cleavage is predominantly mediated by activated caspase-2. We also observed an obvious distribution change of INF2 and caspase-2 in infected cells, indicating their participation in viral ROs formation. The results also suggest that caspase-2 mediated INF2 cleavage is a positive feedback regulatory mechanism of EV71-induced mitochondrial disorders in infected cells (Figure 8), which provides clues for further elucidating the pathogenesis of EV71.

Our study investigated the activation state of human caspase-1–10 in EV71-infected cells and found that multiple caspases were activated. Using CRISPR-Cas9 mediated gene knockout technology, we identified caspase-2 as the main player that cleaved INF2 eventually. In a previous study, Julien et al. (2016) used quantitative mass spectrometry to determine hundreds of natural protease substrates’ catalytic efficiencies in the cell lysate of caspase -2 and caspase -6. INF2 was identified as a substrate of caspase-2 at that time (Julien et al., 2016). However, this study is an *In vitro* cleavage assay. It is not clear whether INF2 could be cleaved in cells under physiological conditions or specific stimulus. To our knowledge, this work represents the first evidence showing that INF2 could be cleaved by activated caspase-2 in living cells, indicating that caspase-2-mediated INF2 cleavage may have some biological functions. As for caspase-2, it has been known for many years as a functionally redundant initiator caspase. It has been implicated in both apoptotic and non-apoptotic signaling pathways, but there is still no clear functional role for caspase-2 due to the poorly identified cellular substrates and inadequate new technologies to investigate its distinct activation pathways (Lopez-Cruzan et al., 2016; Vigneswara and Ahmed, 2020). Our study not only identified a new cellular substrate of caspase-2 but also suggests a new role of caspase-2 in viral replication. However, whether this role is related to other reported functions of caspase-2 should be elucidated carefully in the future.

INF2 is a formin protein that can regulate both polymerization and depolymerization of actin filaments. Recent studies have demonstrated that INF2-mediated actin polymerization stimulates the dynamics of both the outer mitochondrial membrane (OMM) and the inner mitochondrial membrane (IMM) division through Drp1 recruitment and increased matrix calcium, respectively (Korobova et al., 2013; Ji et al., 2015; Manor et al., 2015; Chakrabarti et al., 2018; Steffen and Koehler, 2018). However, INF2 is an ER-bound protein. ER-mitochondrial contacts are required for the regulatory role of INF2 on mitochondria (Steffen and Koehler, 2018). Our previous studies have shown that mitochondria and ER are involved in EV71 replication and suggested that EV71-induced ROs originate from the ER since we observed that large quantities of membranous vesicles accumulated in the perinuclear region in infected cells (Wang et al., 2017). In this study, the distribution change of INF2 and caspase-2 in the perinuclear region in infected cells was also observed. Among them, INF2 signals were attenuated and replaced by some large punctate structures in the perinuclear region, while caspase-2 formed aggregation foci and co-localized with EV71 2C in this region. We speculate that the cleavage of INF2 participates in EV71-induced ER and mitochondrial disorders and is engaged in the membrane structure re-arrangement to form viral-induced ROs. However, when INF2 is knocked out, viral replication was not affected. We suspect that INF2 is functionally redundant with other proteins. A similar phenomenon was observed with other molecules, such as the Golgi brefeldin A-resistant guanine nucleotide exchange factor1 (GBF1) reported in our previous study (Wang et al., 2017). It redistributed to viral ROs but did not affect viral replication when it is silenced down.

In a word, we demonstrated that INF2 is cleaved by activated caspase-2 in EV71-infected cells. This cleavage is related to mitochondrial disorders in infected cells and may involve the formation of viral ROs. This study provides new clues for elucidating the pathogenesis of EV71 and developing potential targets of antiviral treatment of EV71 infection.

## Data Availability Statement

The raw data supporting the conclusions of this article will be made available by the authors, without undue reservation.

## Author Contributions

BW, CZ, CY, YZ, and QT performed the experiments. BW and HH wrote the manuscript. HH and ZZ conceived the project and provided overall direction. All authors contributed to the article and approved the submitted version.

## Conflict of Interest

The authors declare that the research was conducted in the absence of any commercial or financial relationships that could be construed as a potential conflict of interest.
