# Supplementary material for: Enterovirus 71 Induces INF2 Cleavage via Activated Caspase-2 in Infected RD Cells
Source: Front Microbiol. 2021 May 11;12:684953. doi: 10.3389/fmicb.2021.684953 (PMC8144320; doi:10.3389/fmicb.2021.684953)
Supplement: Supplementary file 1 [file Data_Sheet_1.docx]

## Supplementary Materials

## Supplementary Materials and Methods

**N-terminal amino acid sequencing**

RD cells were transfected with plasmids encoding INF2-C (925-1249); 24 h after transfection, the cells were mock-infected or infected with EV71 (MOI = 10) for an additional 12 h. The cells were lysed, and Anti-Flag M2 affinity gel (Sigma, A2220) was used to Immunoprecipitated Flag-tagged proteins. Immunoprecipitant complex was then electrophoresed on 11% SDS-PAGE gels, transferred to polyvinyl amine difluoride (PVDF) membranes, and stained with Ponceau S. N-terminal sequencing was performed on the 38 kDa cleavage fragment in EV71 infected cells. The first 10 amino acids of the N-terminal sequences were determined by the Edman degradation method with a PPSQ-33A automatic Sequencer (SHIMADZU, Japan) at Sangon Biotech (Shanghai, China).

## Supplementary Figure Legends

**Figure S1** **(A)** Schematic diagram showing the results of N-Terminal amino sequencing of ten amino acids of the cleavage fragment in EV71-infected INF2-C (925-1249)-transfected RD cells. **(B)** Sequencing data of (A).

**Figure S2** RD cells were transfected with control siRNA or siRNA against caspase-2, -3, -4, -6, -7, -8 and -10, 36 h after transfection, the cells were mock-infected (EV71-) or infected with EV71 (MOI = 10) for an additional 12 h. Then, the cells were harvested, and a Western blot was performed to detect INF2, the corresponding caspases, actin, and VP1. Red asterisk indicated the group with reduced INF2 cleavage.
