# Supplementary figures and images for: Enterovirus 71 Induces INF2 Cleavage via Activated Caspase-2 in Infected RD Cells
Source: Front Microbiol. 2021 May 11;12:684953. doi: 10.3389/fmicb.2021.684953 (PMC8144320; doi:10.3389/fmicb.2021.684953)

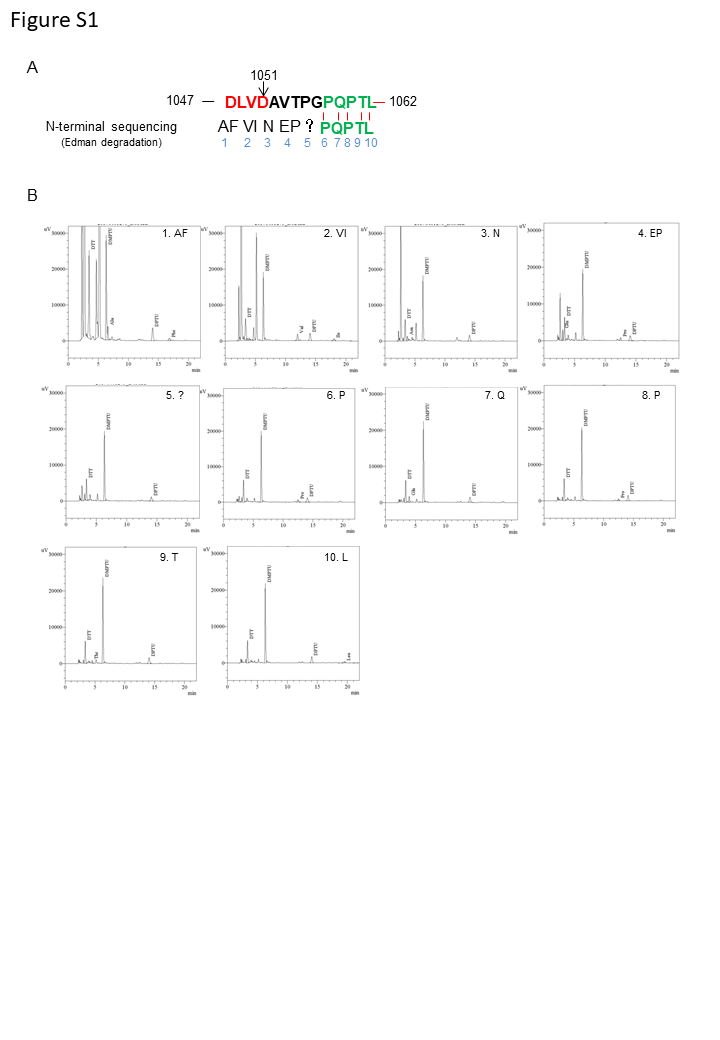

Supplement: Supplementary file 2 [file Image_1.tif]

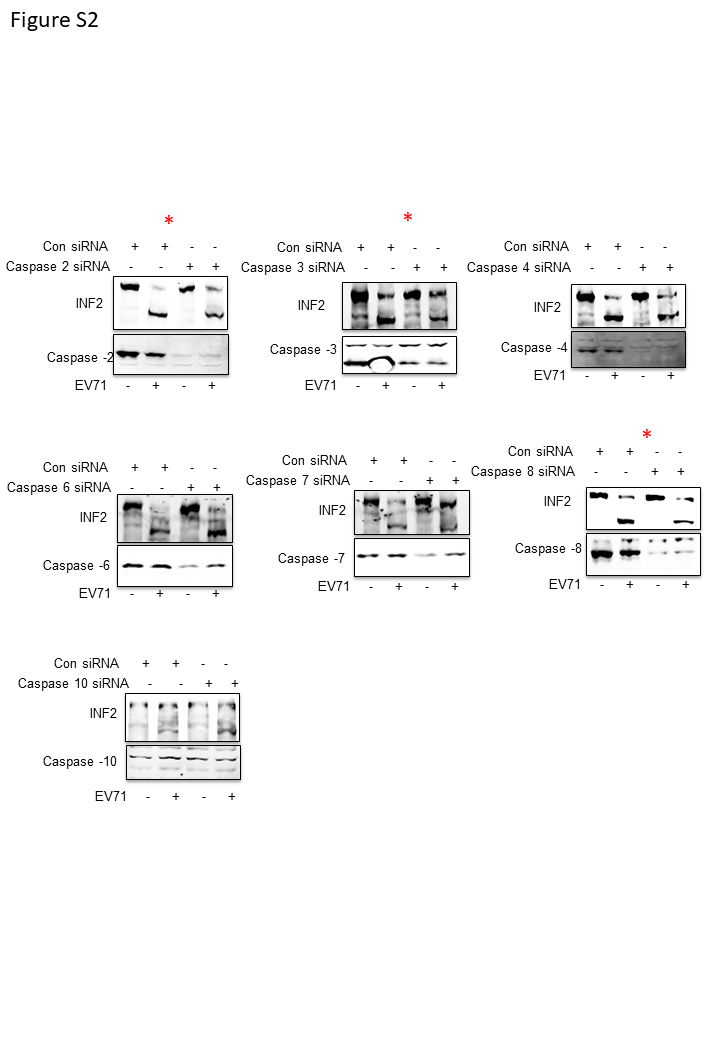

Supplement: Supplementary file 3 [file Image_2.tif]
